# A Trust-Based Predictive Model for Mobile Ad Hoc Network in Internet of Things †

**DOI:** 10.3390/s19061467

**Published:** 2019-03-26

**Authors:** Waleed Alnumay, Uttam Ghosh, Pushpita Chatterjee

**Affiliations:** 1 Computer Science, King Saud University, P.O. Box 2455, Riyadh 11451, Saudi Arabia; wnumay@ksu.edu.sa; 2 Electrical Engineering and Computer Science, Vanderbilt University, Nashville, TN 37235, USA; 3 Department of Modeling, Simulation and Visualization Engineering, Old Dominion University, Norfolk, VA 23529, USA; pushpita.c@gmail.com

**Keywords:** MANET, security, trust, ARMA, GARCH, clustering, IoT, predictive

## Abstract

The Internet of things (IoT) is a heterogeneous network of different types of wireless networks such as wireless sensor networks (WSNs), ZigBee, Wi-Fi, mobile ad hoc networks (MANETs), and RFID. To make IoT a reality for smart environment, more attractive to end users, and economically successful, it must be compatible with WSNs and MANETs. In light of this, the present paper discusses a novel quantitative trust model for an IoT-MANET. The proposed trust model combines both direct and indirect trust opinion in order to calculate the final trust value for a node. A Beta probabilistic distribution is used to combine different trust evidences and direct trust has been calculated. The theory of ARMA/GARCH has been used to combine the recommendation trust evidences and predict the resultant trust value of each node in multi-step ahead. Further, a routing protocol has been designed to ensure the secure and reliable end-to-end delivery of packets by only considering trustworthy nodes in the path. Simulation results show that our proposed trust model outperforms similar existing trust models.

## 1. Introduction

The Internet of things (IoT) is a heterogeneous network of physical devices, vehicles, home appliances, and other objects embedded with electronics, software, sensors, actuators, and connectivity. By enabling seamless connection between objects and the rapid exchange of large volumes of data, this paradigm offers a new class of advanced services characterized by being available irrespective of time, place, and person. It may comprise different types of wireless networks such as wireless sensor networks (WSNs), ZigBee, Wi-Fi, mobile ad hoc networks (MANETs), and RFID. The deployment of IoT devices grew significantly in present years. This includes buildings (homes, schools, offices, factories, etc.), utility networks (electricity, gas, water, etc.), transportation networks (roads, railways, airports, harbors, etc.), transportation vehicles (cars, rails, planes, etc.), healthcare systems, information technology networks, and so on. The use of wireless networks makes the physical infrastructures more smart, secure, reliable, and the systems become fully automated. IoT collects, stores, and exchanges a large amount of heterogeneous data from different types of networks and provides critical services in healthcare, manufacturing, and utility networks.

Mobile Ad hoc Network (MANET) is a distributed collection of wireless nodes which can work without the presence of any centralized administration or fixed network infrastructure. In this network, the nodes are free to move randomly at any given time. The mobility of nodes can vary from almost stationary to constantly moving nodes. Thus, the topology of the network and interconnections between the nodes can change rapidly and unpredictably. In MANET, the nodes within the radio range can immediately communicate with each other, whereas the nodes that are not within each other’s radio range are able to communicate with the help of intermediate nodes where the packets are relayed from source to destination. Therefore, the nodes in a MANET have to co-operate among themselves for the multi-hop communications.

Security is a prime issue in MANET due to weak connectivity, resource constraints, and limited physical protection of the mobile nodes. Therefore, MANETs are more prone to attacks than infrastructure-based networks. Establishing secure communication in a MANET is particularly challenging because of (i) shared wireless medium; (ii) no clear line of defense; (iii) self-organizing and dynamic network; (iv) most of the messages are broadcasted; (v) messages travel in a hop-by-hop manner; and (vi) nodes are constrained in terms of computation and battery power.

Trust can play an important role to improve security of ad hoc networks by a-priori or run-time evaluation of trustworthiness of its peers before making any routing decision. Due to resource (e.g., energy, bandwidth) constraints, all the nodes may not take part in routing and subsequently they do not forward data packets. To ensure the availability of the nodes and provide secure end-to-end communications between them, the trustworthiness of a node is a good measure. Cryptographic mechanisms cannot help in order to detect/prevent such random behaviors which pose security threats to the network. The notion of trust management does not replace cryptography, but rather supplements to it. Cryptography and a trust decision framework can work hand-in-hand to achieve holistic security in IoT-MANET.

In order to ensure reliable, secure, and timely delivery of packets in the network, the present work has designed and developed an evidence/behavior-based trust framework in a cluster-based IoT-MANET. The nodes of the IoT-MANET are grouped into one-hop clusters [1,2] and a predictive trust management model is developed for trust calculation and propagation. This model is well suited for detecting malicious nodes and improving the performance of the network.

Trust is sociological concept first defined by Gambetta [3]. The term trust has been coined in the social science literature and adopted by distributed computing and mobile computing as well. Trust is a combination of opinion, belief prediction, and probability [3]. Trust is a measurement of reliability, utility, and availability, and it also improves the overall network functionalities like quality of services, reputation, availability, risk, and confidence [4].

Trust quantification and evaluation has been fostered in recent years for a wide range of applications. As discussed above, trust is a function of uncertainty which incurs the level of confidence about a node for a particular activity. If the node believes that its peer would definitely not perform some action in any circumstance, the node does not “trust” the latter, again if it believes it would complete the particular task, the trust level is high. In both cases there is no uncertainty. But if the node is not confident about the next move of the other then uncertainty occurs. Unfortunately, nodes involved in IoT-MANET show this kind of uncertainty in their behavior. In the present proposal, the level of trust can be measured by a continuous real number, referred to as the *trust value*. Trust is not necessarily symmetric. The fact that A trusts B does not necessarily mean that B also trusts A, where A and B are two entities. As trust is subjective probability [4], when a system converges to a trusted stage it is not well addressed. This paper deals with a distributed scheme to utilize trust evidences for secure data delivery in IoT-MANET. The trust-based framework is based on the notion of point-to-point trust to prevent malicious behaviors of MANET nodes.

(a) **Contribution:** This paper discusses an evidence-based quantitative trust management scheme that supports both direct and indirect trust opinion. The direct trust is calculated using a quantitative model by taking consideration of trust parameters which are combined by β distribution. For calculating final or resultant trust, various recommendations are collected from other common neighbors, and combined using a ARMA/GARCH mathematical model of prediction [5]. Using the notion of multilevel prediction, trust is quantified with good accuracy. Through extensive simulation, the model is evaluated and compared with an existing trust management scheme [1]. The proposed model is lightweight in terms of computation and powerful in terms of flexibility and accuracy in managing trust in IoT-MANET.

(b) **Organization of the paper:** The rest of the paper is organized as follows: Section 2 gives a brief note on the related research efforts in the area of trust and secure routing in MANET. In Section 3, we present our proposed trust model. Section 4 presents the simulation results of the proposed trust protocol along with existing protocols. Section 5 discuss about the security analysis, and finally conclusions are presented in Section 6.

## 2. Related Work

MANET has been well studied since the last decade. In recent years, the proliferation of the internet of everything concept increases the use and utility of MANET significantly. IoT-MANET is a budding topic because devices are wirelessly connected and most of them may be energy constrained, and networks are self-organized. Therefore, security and reliability requirements of such networks need to be re-investigated. Several trust-based routing protocols have been designed and evaluated in an ad hoc networking scenario. In the present section the state-of-the-art study has been summarized. Trust management schemes for MANET are the central point of attraction. We also discuss secure routing and trust propagation as well.

Most reputation-based trust management schemes are devised for collaborative secure routing by detecting misbehaving nodes that are either selfish or malicious. Upon designing secure routing protocols, researchers assumed a-priori trust relationships between mobile nodes. Wang et al. proposed a mechanism [6] to distinguish selfish peers from cooperative ones based solely on local observations of ad hoc on-demand distance vector (AODV) routing protocol. They use a finite state machine model of locally observed AODV actions to construct a statistical description of each peer’s behavior. An interesting extension of this work would be to consider various patterns of node mobility, which can give additional insights. Virendra et al. proposed a trust-based security architecture [7] for key management in WANETs. The unique part of this work is that it considers the trust level of each node in a physical as well as a logical sense. Jiang and Baras [8] addressed distributed trust computation and establishment using random graph theory and the theory of dynamic cooperative games. Liu et al. proposed an extension of AODV-based routing protocol B-AODV [9], where trust plays an important role to secure end-to-end delivery. Velloso et al. proposed a trust management scheme [10] for a proposed human-based model for establishing trust relationships between the nodes of an ad hoc network where local trust ratings are sufficient for calculating trust. They showed that their proposed scheme required a minimum number of messages for a large global network. In [11,12], Jain et al. proposed a trust-based framework to mitigate wormhole attack in an ad hoc network scenario. In extension to their work, Jain et al. proposed an extension of AODV by incorporating a quantitative trust model [13], which takes different metrics from the physical and application layers to provide a secure routing solution.

In [14], Zhang et al. proposed a formal analysis of a trust-based routing method that used routing algebra and group-based trust computation. In [15], Desai et al. proposed a predictive routing model where they identified the detect sequence number attacks. Kaur et al. discussed the performance of AODV, DSR, and ZRP under the impact of multiple wormhole attacker nodes in [16]. Chatterjee et al. proposed a number of distributed, secure, trust-aware clustering protocols [17,18,19,20,21] in order to provide secure solution for end-to-end data delivery. In [22], Xu et al. presented a mathematical framework and analyzed the routing protocols in order to find the efficacy of the algorithms. In [23], Rath et al. described major safety and security related issues for MANET connected Internet of Things (IoT). They focused on the vulnerability and security threat for ubiquitous computing and IoT in light of ad hoc network formation on the fly. A privacy-preserving and secure link-state based routing protocol (ALARM) has been proposed by Defrawy et al. in [24]. They demonstrated the capability of ALARM to achieve privacy and security, and anonymity, authentication, integrity as well. In [25], Defrawy et al. designed a secure routing protocol to evade both outsider and insider adversaries. They claimed that the proposed reactive location-aware anonymous MANET routing protocol, namely PRISM, can achieve better privacy and security and outperform ALARM. From the state-of-the-art study, it is relevant that a better prediction model for trust computation is necessary to achieve reliable and secure data delivery in IoT-MANET.

## 3. Proposed Protocol

A prediction-based trust management framework has been made to enable nodes to establish a trustworthy route and reliable data delivery in IoT-MANET. We have adopted the clustering framework as described in [26] for clustering and Clustrehead (CH) election. In order to collect network parameters for trust calculation, node *A* monitors traffic of each neighbor of node *B* and calculates the direct trust evidence $\left(\zeta_{t}^{AB}\right)$ in a time period $\tau_{Trust}$. Nodes are categorized from their *Good* and *Bad* behaviors. To ensure reliable data delivery, the following network parameters have been considered;

1. number of packets properly forwarded (*Good*)
2. number of packets dropped (*Bad*)
3. number of packets falsely injected (*Bad*)

Trust calculations have been performed periodically, and after the expiration of each time period of $\tau_{Trust}$, the trust parameters are collected and direct observation $\zeta_{dir}$ is calculated by node *A* using a β distribution.

### 3.1. The Trust Model

**Figure 1:**
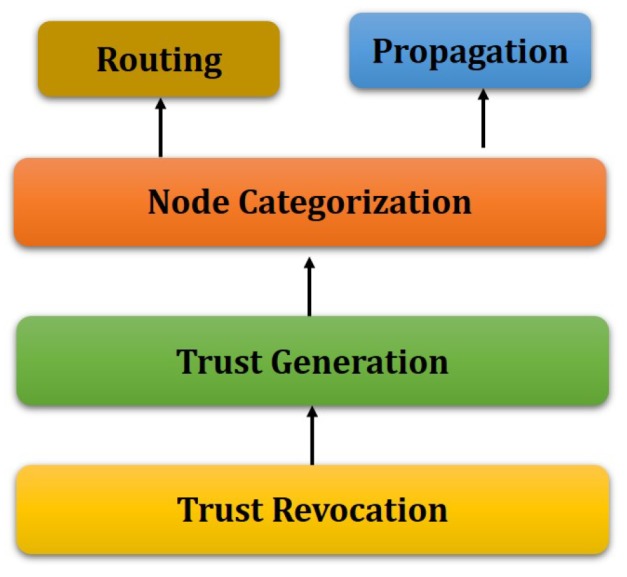
The trust model

**Figure 2:**
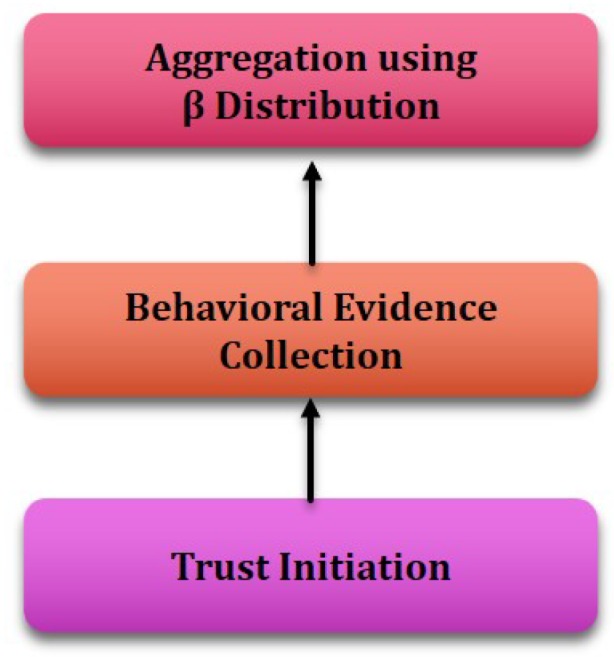
Trust generation

**Figure 3:**
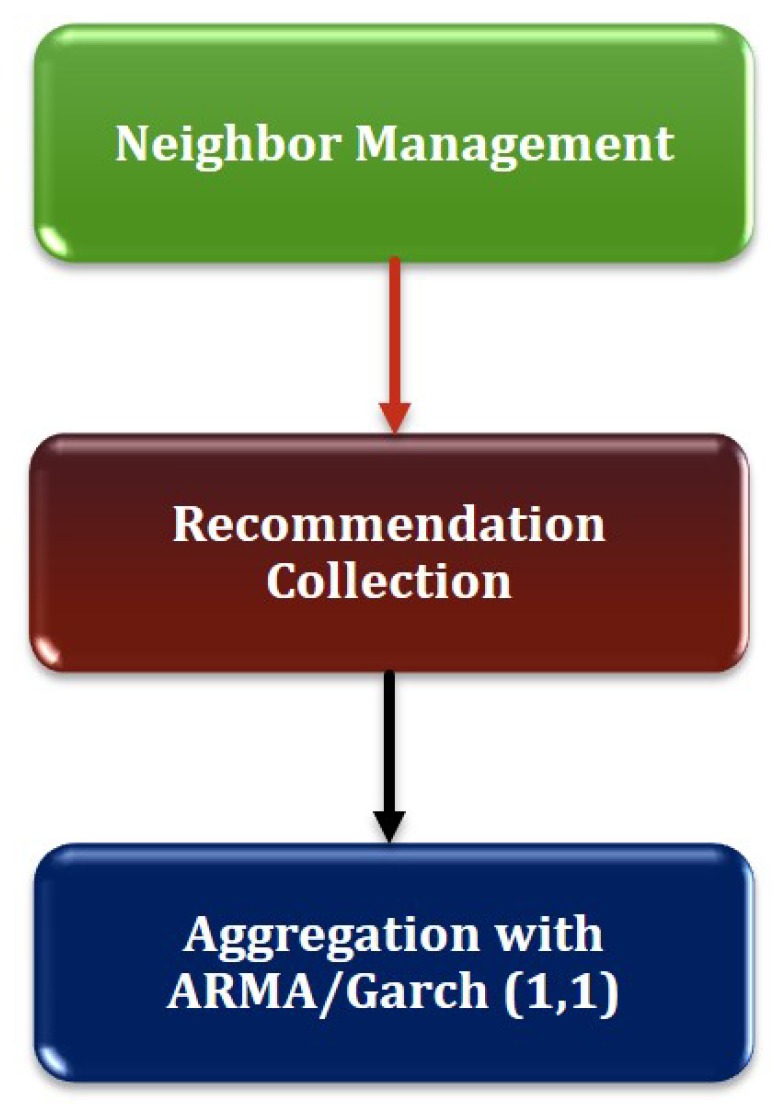
Resultant trust generation

Figure 1 shows the system architecture of the proposed model. The trust generation model is presented in Figure 2 and Figure 3. As depicted in Figure 1, the Clusterhead initiates the trust calculation and takes responsibility of trust propagation and establishing routes from source to destination in order to achieve reliable end-to-end delivery of packets and node availability.

1. **Trust Revocation:** This is the first phase when CH initiates the trust calculation of each member of the cluster. Due to the energy-constrained nature of IoT-MANET nodes, it is not appropriate to calculate trust at fixed intervals. Similarly, singleton trust generation is not good as the nodes are vulnerable to uncertain behavior due to the quasi-static nature (node mobility). The trust model is evidence-based, and it may change from time to time. Therefore, a trade-off between the trust revocation and trust initialization is required. This manager assigns an aging factor to each trust value, which fades the trust values. This factor is application-specific and it can be set depending upon the requirement.
2. **Trust Generation:** In this phase, the trust value of each member of a cluster is evaluated. This phase is performed in two separate phases; direct trust ($\zeta_{dir}$) calculation and resultant trust ($\zeta_{res}$) generation. The detail procedure is described below. After calculating the trust value nodes are categorized.
3. **Node Categorization:** In MANET, a packet can be dropped by a node due to network congestion, link failures (node mobility) between nodes, network interference and contentions, selfishness (the nodes having limited energy saves their energy by not forwarding the packets), and maliciousness (the malicious nodes intentionally do not forward the packets). In an ideal scenario, the packets are dropped only due to maliciousness of a node. However in a real scenario, there are some inherent properties of the medium for which packets may be dropped, though the actual reason cannot be identified easily. Therefore, we define a “bad node” as a node that randomly drops packets deliberately. In our scenarios packets will only be dropped for that reason, and not for other intrinsic network reasons (congestion, link failures, etc.). In this node categorization phase, CH categorizes the member nodes into groups for their good behavior and bad behavior using a threshold limit between $Max_{\mathrm{TH}}$ and $Min_{\mathrm{TH}}$. These threshold values can be set according to the network deployment scenarios.
  - *Good:* if $\zeta_{res}\geq Max_{\mathrm{TH}}$;
  - *Bad:* if $\zeta_{res}\leq Min_{\mathrm{TH}}$;
  - *Uncertain:* if $Min_{\mathrm{TH}}<\zeta_{res}<Max_{\mathrm{TH}}$.
4. **Trust Propagation:** Once the resultant trust of each member node is finalized, in this phase CH propagates these values to the other cluster members.
5. **Routing:** For creating routes between source and destination, maintaining the routes and the network, the proposed routing needs to perform the following three phases.
  - **Neighbor Discovery:** This phase is responsible for maintaining the list of trusted neighbors, along with their trust status.
  - **Route Discovery:** In this phase the end-to-end path is established by including only good and uncertain nodes.
  - **Route Maintenance:** This phase maintains the established route. Each node on an active path monitors the link periodically. It also revokes the route discovery phase if link failure occurs due to node mobility.

### 3.2. Trust Generation

Trust generation has been achieved in two steps; direct trust calculation and resultant calculation.

#### 3.2.1. Direct trust calculation

1. **Trust Initiation:** Along with bootstrapping the IoT-MANET, CH and cluster members initiate the trust calculation. Once the trust value has aged, the trust revocation procedure is restarted and the direct trust calculation takes place.
2. **Evidence Collection:** In this phase both CH and cluster members collect the *Good* and *Bad* evidences, as described in Section 3.
3. **Trust Aggregation:** In this phase each member and CH of a cluster collects and stores collective data for all good and bad events. A reputation system-based on the β probability density function has been used, in order to calculate direct trust. The β function represents probability distributions of binary events (either good or bad). It is a mathematical method for combining feedback and expressing reputation ratings. A brief description of β distribution is presented in Appendix A.

Suppose that node *X* has collected information about positive (good) and negative (bad) behaviors about node *Y*. Good behavior is represented by α, and β represents bad behavior. The posterior probabilities of trust value can be predicted using the β distribution function, which is presented in Equation (1). The β distribution can be expressed using the Γ function as

(1) $$f\left(p|\alpha ,\beta \right)=\frac{\Gamma (\alpha +\beta )}{\Gamma (\alpha )+\Gamma (\beta )}p^{\left(\alpha -1\right)}{\left(1-p\right)}^{\left(\beta -1\right)}$$

where 0≤p≤1, α,β>0, with the restriction that the probability variable p≠0 if α<1, and p≠1 if β<1.

It is an uncertain probability and the expected probability of trust value is E(p). Therefore, it can be said that the relative frequency of outcome is most likely to be E(p). The direct trust of a node under review can be calculated using Equation (2).

(2) E(p)=α/(α+β)

Each node calculates and stores the trust value about its neighboring nodes. Here, α and β correspond to the accumulated number of good behavior and bad behavior. Initially, the direct trust value of each node is set to 0.5 as there are no recorded observations or evidences about a node under review. Thus, we start with α=β=1, which makes both the values of expectation and the direct trust equal to 0.5. Here, we train the Beta function according to [27].

#### 3.2.2. Resultant Trust Calculation

1. **Neighbor Management:** Each member of a cluster (including CH) maintains a list of neighboring nodes. CH broadcasts *Hello* messages periodically and maintains a list of node-IDs of members in the cluster. Each member also exchanges *Hello* messages periodically to keep track of its neighbors.
2. **Trust Information Collection:** Here, CH collects the recommendation from common neighbors to compute the resultant trust of a node under review.
3. **Trust Aggregation:** On receiving the recommendation of trust evidences from the members, CH executes ARMA(1, 1)/GARCH(1, 1) to calculate the resultant (or final trust) of a node under review. The resultant trust of a node can be calculated using ARMA(1,1), as shown in Equation (3).
  (3) $$X_{t}=\varepsilon_{t}+\Sigma_{i=1}^{p}\varphi_{i}X_{t-i}+\Sigma_{i=1}^{p}\theta_{i}X_{t-i}$$

To estimate $\varepsilon_{t}$, which is independent of *s* (where *s* is set of independent trust evidences collected from common neighbors and self-evidence also), we adopt normal distribution with zero mean and constant variance $\sigma_{\varepsilon }^{2}$. So, the likelihood function for the ARMA (1,1) model [5] is computed by the following equation.

(4) $$\begin{array}{c} L\left(\varphi_{1}\theta_{1},\sigma_{\varepsilon }^{2}\right)=\prod_{t=2}^{T}\frac{1}{\sqrt{2\Pi }\sigma_{\varepsilon }}\\ \times \exp\{-\frac{{\left(y_{t}-c-\varphi_{1}y_{t-1}-\theta_{1}\varepsilon_{t-1}^{*}\right)}^{2}}{2\sigma_{\varepsilon }^{2}}\} \end{array}$$

Therefore, the log likelihood function is

(5) $$\begin{array}{c} l\left(\varphi_{1}\theta_{1},\sigma_{\varepsilon }^{2}\right)=-\left(T-1\right)\log\sigma_{\varepsilon }-\frac{1}{2\sigma_{\varepsilon }^{2}}\\ \times \sum_{t=2}^{T}{\left(y_{t}-c-\varphi_{1}y_{t-1}-\theta_{1}\varepsilon_{t-1}^{*}\right)}^{2} \end{array}$$

where $\varepsilon_{t}^{*}=y_{t-1}-c-\varphi_{1}y_{t-2}-\theta_{1}\varepsilon_{t-2}^{*}$ for t=3,…,T are obtained recursively.

The GARCH (1, 1) model is expressed as

(6) $$\sigma_{t}^{2}=k+G_{1}\sigma_{t-1}^{2}+A\varepsilon_{t-1}^{2}$$

where $\sigma_{t}^{2}\varepsilon_{t}$ is taken from from the ARMA(1,1) model and it is assumed that it has conditional variance $\varepsilon_{t}^{2}$. In case of Gaussian $\varepsilon_{t}$, the likelihood function is

(7) $$L\left(k,G^{1},A_{1}\right)=\prod_{t=2}^{T}\frac{1}{\sqrt{2\Pi }\varepsilon_{t}}\exp\left\{-\frac{\varepsilon_{t}^{2}}{2\sigma_{\sigma_{t}^{2}}^{2}}\right\}$$

The log likelihood function, neglecting the constant term, can be written as

(8) $$l\left(k,G^{1},A_{1}\right)=\frac{1}{2}\sum_{t=2}^{T}\left\{\log\sigma_{t}^{2}+\frac{\varepsilon_{t}^{2}}{\sigma_{t}^{2}}\right\}$$

where $\sigma_{t}^{2}=k+G_{1}\sigma_{t-1}^{2}+A_{1}\varepsilon_{t-1}^{2}$ are obtained recursively.

CH can predict the multiple-step ahead value of the trust series using ARMA/GARCH model, therefore finding the likelihood of good nodes has been increased significantly and it also reduces the trust revocation largely. For energy constrained environments like IoT-MANET, this scheme provides flexibility in terms of energy. In the following Section we will examine the efficacy of the proposed model.

## 4. Simulation

To show the efficacy of the proposed trust-based routing protocol we have compared it to CBRP [28] and the protocol proposed by Chatterjee et al. [1]. We have implemented all these routing protocols on top of AODV using the NS-2 (version-2.34) simulator [29].

In [1], a distributed trust model for securing MANETs has been proposed. Here, the direct trust of a node is calculated by monitoring its behavior and collecting information about the node. It chooses different parameters (such as the number of packets forwarded, dropped, misrouted, and falsely injected) for the direct trust calculation. CH periodically collects the direct trust values of member nodes and uses the modified Dempster–Shafer theory [30,31] to compute the global trust value of a node. In our present work, the direct trust is calculated using a quantitative model by taking into consideration the trust parameters which are combined by the β distribution. In order to calculate the resultant trust value of a node under consideration, various recommendations are collected and combined using the ARMA/GARCH model. The proposed trust model can predict the multiple-step ahead value of the trust series using the ARMA/GARCH model.

### Simulation Parameters

In the simulation, the IEEE 802.11 standard has been used as the MAC layer protocol. The transmission range of each node is set to 250 m. The nodes travel with a speed varied from 0 m/s to 5 m/s, with a pause time set to 5 s. A cluster may get partitions very frequently due to high node mobility. All the trust-based protocols, including our proposed protocol, may not be suitable in highly dynamic networks. UDP has been used as the transport layer protocol with constant bit rate (CBR) traffic generator of packet size 512 bytes. Simulation has been performed for 500 s with a 21-node cluster over a network area of 450 m × 450 m. The proposed trust protocol is based on a 1-hop cluster and hence the network area is chosen to be 450 m × 450 m. The malicious nodes drop the packets randomly whereas the good nodes drop the packets due to the environment of the network (such as mobility of nodes, collisions of packets, etc.) in our simulation. It can be observed that around 15%–20% of the packets (non-malicious drops) are lost due to the environment of the MANET. The actual reason for packet drop by a node cannot be identified easily. Thus, we consider a node as a bad node if the trust value reaches $Min_{\mathrm{TH}}$ due to excessive packet drops and other malicious activities by the node. The summary of simulation parameters are shown in Table 1.

**Figure 4:**
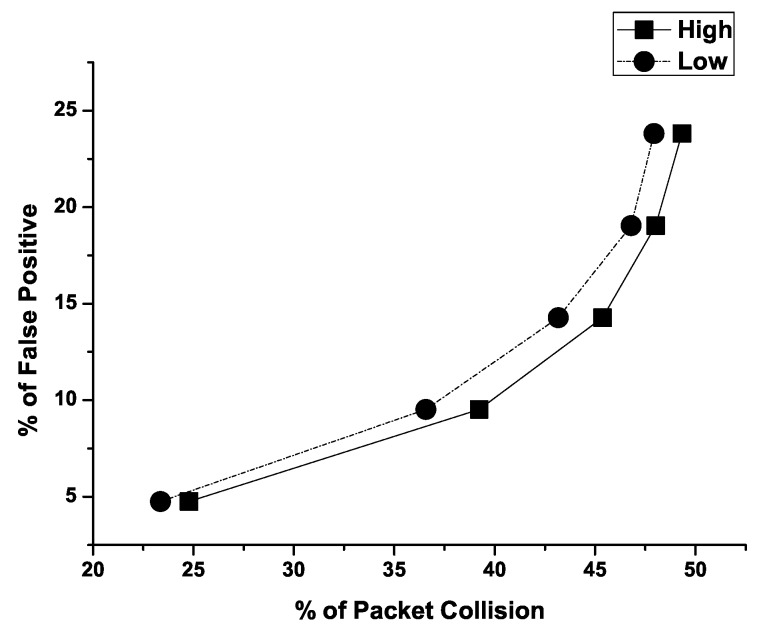
False positive vs. packet collision.

**Detection Ratio: False Positive:** To evaluate the accuracy of the proposed trust model, false positive is chosen as the performance metric. False positive is measured as the ratio of the number of good nodes falsely detected as malicious to the total number of nodes in the network. Figure 4 presents the percentage of packet collision versus percentage of false positive of the proposed trust model. From the graph it is evident that only 5% nodes are wrongly detected as malicious when 24.78% and 23.36% packet collisions occur for *Low* level and *High* level, respectively. Again, the proposed trust model falsely detects nearly 24% nodes as malicious when 49.32% and 47.93% packet collisions take place for *Low* level and *High* level, respectively. Therefore, the proposed trust model performs well even when the rate of packet collision in the network is high. It should be noted that this false detection can be varied by varying the max_threshold and min_threshold parameters. These values are application-specific.

**Performance Evaluation:** We considered four parameters, packet delivery fraction (PDF), packet drop rate (PDropR), packet delivery ratio (PDelR), and throughput (THR), as performance metrics in order to compare the proposed trust-based routing protocol with two existing and well-studied protocols, which are CBRP [28] and the protocol proposed by Chatterjee et al. [1].

**Figure 5:**
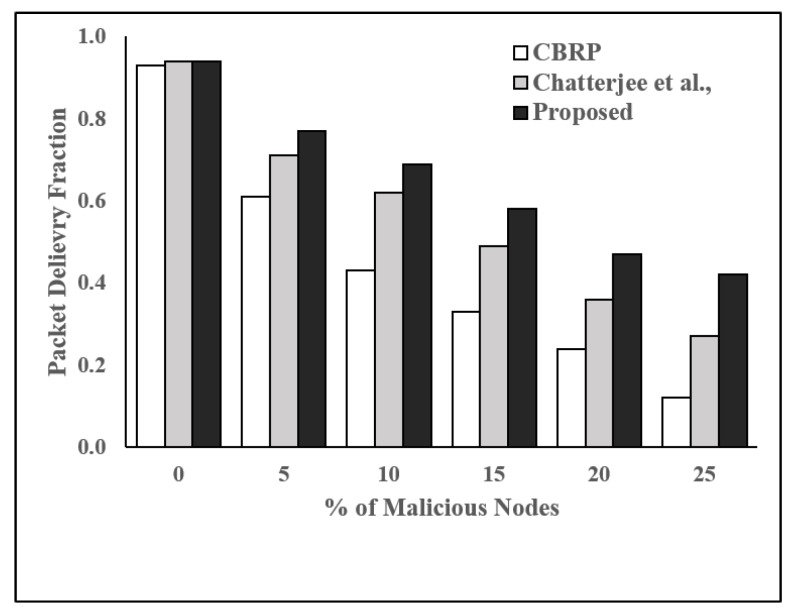
Packet delivery fraction vs. % of malicious nodes.

**Figure 6:**
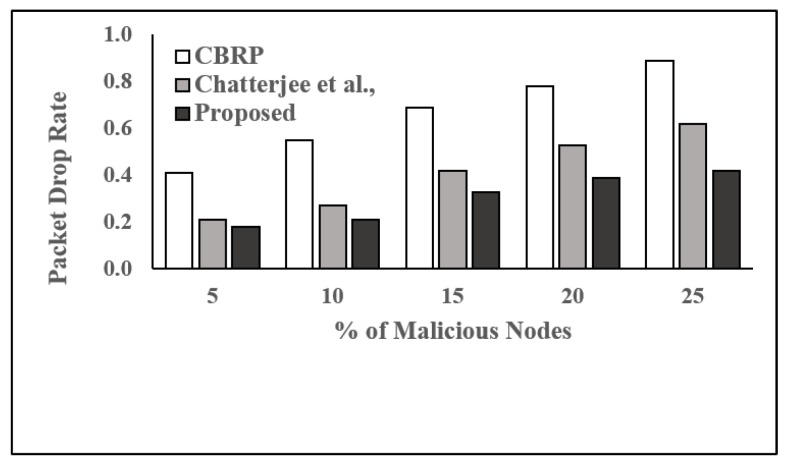
Packet drop rate vs. % of malicious nodes.

**Figure 7:**
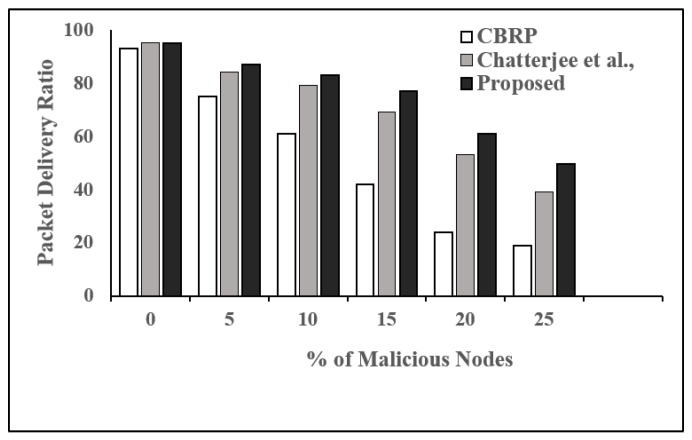
Packet delivery ratio vs. number of malicious nodes.

**Figure 8:**
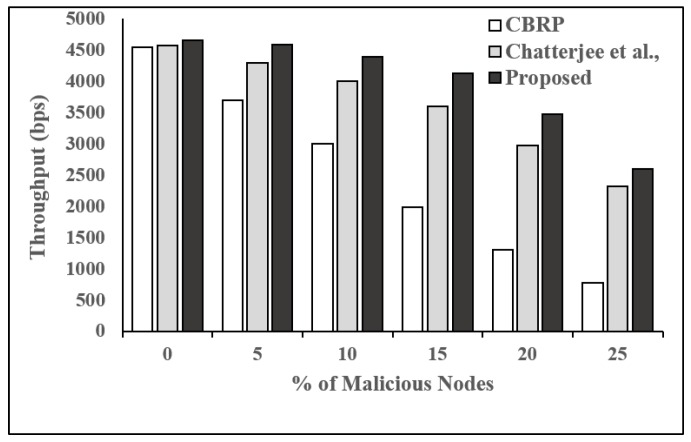
Throughput vs. number of malicious nodes.

Figure 5, Figure 6, Figure 7 and Figure 8 show the effect of malicious node on PDF, PDropR, PDelR, and ThR, respectively for the protocols under consideration. It is evident that the packet delivery fraction is more than 0.9 in the absence of malicious nodes for all schemes. However, PDropR increases and subsequently PDF decreases with the increased number of malicious nodes for all the protocols. CBRP gives 0.61 and 0.12 as PDF when nearly 5% and 25% malicious nodes are present in the network, respectively. The protocol proposed by Chatterjee et al. offers 0.71 and 0.27, whereas our proposed protocol gives 0.77 and 0.42 as PDF for the same percentages of malicious nodes.

From Figure 6, we can see that PDropR increases with the increased number of malicious nodes in the network. In CBRP, PDropR increases from 0.41 to 0.89 when the number of malicious nodes increases from about 5% to 25%. It is evident that the proposed protocol outperforms the protocol proposed by Chattejee et al. and CBRP. This is due to the fact that our proposed protocol can predict the trust value of a node at least one step ahead and provides trustworthy routes between source and destination.

Figure 7 shows the impact of malicious node behavior on average PDelR for CBRP, the protocol proposed by Chatterjee et al., and our proposed protocol. All the protocols give around 93% PDelR when none of the nodes are malicious. Due to the absence of security mechanisms in CBRP, the PDelR reduces to 19% in the presence of 25% malicious nodes. Though Chatterjee et al. consider the security aspects (trust) while setting up the route, the proposed protocol outperforms the other two protocols and gives around 50% PDR even when 25% of the nodes behave maliciously.

From Figure 8, we can observe that all the protocols give good throughput when none of the nodes are behaving maliciously. It can also be seen that the proposed protocol outperforms the other protocols in the presence of malicious nodes. In the presence of 25% malicious nodes, the proposed scheme gives throughput around 2600 bps whereas CBRP and the protocol proposed by Chattrejee et al. offer throughput around 800 bps and 2300 bps, respectively.

## 5. Security Analysis of the Protocol

From the simulation results presented in the previous section, it can be seen that the proposed protocol outperforms the similar routing schemes. In this section, we discuss the attack model for the proposed protocol. The trust model runs in real time and the malicious (i.e., dropped or falsely injected packets) can be detected on-the-fly and isolated as a *blacklisted* node.

- *Address Spoofing:* In this attack, the attacker tries to spoof an address (that is IP and ID) of a victim node. Using a one-way secure hash function the present scheme can prevent the attacker from stealing the ID/IP of a node. Moreover, the attacker cannot generate the signature of the victim node. This is because an attacker may know the public key, but it is difficult to know the private key of the victim’s node.
- *Routing Table Overflow and resource consumption attacks:* In the proposed model, trust is calculated real time and nodes are being monitored for their functionality in order to calculate trust. Thus, any compromised or malicious attacker generating overflow or resource consumption can be detected.
- *Byzantine/Blackhole/DoS Attack:* During node monitoring, if a node finds another neighbor sending packets to a particular node repeatedly, dropping packets, or holding the packet more for than a certain interval, a *Warning* message is generated and temporarily restricts other good nodes from communicating with the suspicious node until the next review comes. In this way, Byzantine/blackhole/DoS attacks can be detected and prevented.
- *Sleep Deprivation Attack:* In a similar manner, generating unnecessary traffic (e.g., seeking some information repeatedly in a small interval of time) can be identified and a *Warning* message is generated. Unless it behaves properly, any request from that node will not be entertained. Thus, sleep deprivation attacks may be identified and mitigated.

In the proposed scheme, nodes need a certain time for bootstrapping to be a member of a cluster. It should be noted that this bootstrapping time is a vulnerable time for the presented protocol. As it is a quantitative model, unless some behaviors are recorded, trust cannot be established. Thus, the proposed scheme requires *vulnerable time* to detect any malicious activities.

## 6. Conclusions

In this paper, a novel quantitative trust model for a clustering environment in IoT-MANET has been proposed. The prediction-based trust model can collaboratively compute the resultant trust of a node using direct trust and recommendation trust opinions from other nodes. The proposed evidence-based trust model utilizes the probabilistic model of β distribution to calculate the direct trust of a node under review. From the resultant or final trust of the node under review, the theory of ARMA/GARCH has been used to predict the future behavior of the node, which is derived from its past behavior. Using a weighted combination model, the direct trust evidence and collected recommendation evidences are combined so that the effect of malicious reporting can be minimized. Extensive simulation study shows that the model outperforms the similar trust-based protocols in terms of false positive detection, even for a highly congested network. The routes have been established between source and destination only including the trusted nodes in the path. Simulation has been carried out to compare the efficacy of the scheme and the results are very satisfying in terms of packet delivery fraction, packet drop rate, packet delivery rate, and throughput compared to clustering and trust-based protocols.

In the future, we will set up a testbed of 10 laptops and implement our proposed trust-based model to validate the simulation results. The proposed trust model can be applied in IoT-WSN and subsequently in smart cities to provide security. Further, it can provide security in software-defined mobile ad hoc networks (SD-MANET), where SDN controllers can collect the recommendation trust and compute the resultant trust value of their nodes.

## Figures and Tables

**Table 1:** Simulation parameters and environment.

Simulation Parameter	Assigned Value
Application Agent	CBR
Packet Size	512 bytes
Transport Agent	UDP
Routing Protocol	AODV
Network area	450 × 450
No. of nodes	21
No. of malicious nodes	5
Mobility Model	Random Way-Point
Addressing Scheme	IDDIP
Mobility	0–5 m/s
Pause Time	5 s
Simulation Time	500 s

## Appendix A. Beta Distribution

The Beta (α,β) distribution is a probability distribution that models events which are constrained to take place within an interval defined by a minimum and maximum value. It is generally a continuous probability distribution that is defined on the interval of (0,1) 0<p<1. Its probability density function can be presented using the following formula: $\frac{p^{\left(\alpha -1\right)}{\left(1-p\right)}^{\left(\beta -1\right)}}{B(\alpha ,\beta )}$, where α and β are two positive shape parameters which control the shape of the distribution, p is a random variable and B(α,β) can be calculated using the following equation:

(A1) $$B\left(\alpha ,\beta \right)=\frac{\Gamma (\alpha )+\Gamma (\beta )}{\Gamma (\alpha +\beta )}$$

where Γ(k)!=1×2×3×…×(k−1).

The expected value of a Beta-distributed random variable *p* can be computed using the following equation:

(A2) $$E\left(p\right)=\frac{\alpha }{\left(\alpha +\beta \right)}$$

The variance of a Beta-distributed random variable *p* can be calculated using the following equation:

(A3) $$V\left(p\right)=\frac{\alpha \beta }{\left(\alpha +\beta +1\right){\left(\alpha +\beta \right)}^{2}}$$

The β distribution is very simple and flexible to use. This is because the distribution has only two parameters (α, β) that for the random variable (*p*) take values between 0 and 1. Consequently, it can often be used to model percentage or fractional quantities. In general, the β distribution can be used a lot in Bayesian statistics in any case where we may have an unknown probabilities of events taking place. Examples of network related events that may be modeled by β distribution include:

- Trust value of a node.
- The number of packets successfully delivered from source-to-destination.
- Node mobility in a wireless network.
- The time it takes for delivering of packets from source-to-destination.
